# Do Psychological Ownership and Communicative Presence Matter? Examining How User-Generated Content in E-Commerce Live Streaming Influences Consumers’ Purchase Intention

**DOI:** 10.3390/bs14080696

**Published:** 2024-08-11

**Authors:** Nan Zhang, Wen Hu

**Affiliations:** School of Computer and Information Engineering, Harbin University of Commerce, Harbin 150028, China; 300017@hrbcu.edu.cn

**Keywords:** live streaming marketing, user-generated content, emotions, psychological ownership, communicative presence, consumer behavior

## Abstract

E-commerce live streaming has become a lucrative global industry. As the main carrier to convey information in live broadcasting, user-generated content (UGC)—and especially bullet screens—are crucial in influencing users’ purchase intentions. However, the influence of bullet screens’ multidimensional information characteristics on consumers’ decision-making processes requires further exploration. Additionally, most existing studies start with the short-term effects of live product realization, and must address how to enhance customers’ psychological ownership using new means of live streaming marketing to obtain long-term sustainable brand-building effects. This study introduces psychological ownership and the communicative presence as mediating variables based on the theory of elaboration likelihood modeling to explore the mechanism of the influence of UGC’s multidimensional features on viewers’ purchase intentions in live e-commerce broadcasting rooms. We collected 404 valid online questionnaires and tested our hypotheses using structural equation modeling. These findings indicate that UGC emotions, quality, and their interaction significantly and positively affect purchase intentions. Moreover, psychological ownership and the communicative presence mediate UGC’s effect on purchase intentions. These results provide a new perspective for understanding consumer behavior in live e-commerce to improve marketing effectiveness of e-commerce live streaming platforms.

## 1. Introduction

Over the past three years, e-commerce live streaming has become a lucrative global industry [1]. China’s live streaming sales surpassed RMB 2.2 trillion between January and October 2023, an increase of 58.9%, making up 18.1% of online retail sales [2]. In the same year, Oriental Selection’s annual gross merchandise volume reached RMB 10 billion, with that of Douyin, a popular Chinese short-video app, accounting for the vast majority [3]. To access the platform, consumers must click the Douyin app on their cell phones. They can then create and share short videos for viewing, liking, and commenting, promoting social interaction. Alternatively, they can join live broadcasting rooms to watch streams that match their interests. By clicking the shopping cart icon, consumers can view all the items available for sale in the live room. They then have the option to either collect or purchase the goods. This shows that it is crucial to understand the uniqueness of e-commerce live streaming and explore the relationship between e-commerce live streaming and viewers’ purchasing behaviors.

E-commerce live broadcasting is characterized by real-time interactions among multiple subjects, which allow consumers to access dynamic, up-to-date, and immersive products, along with relevant clues. E-commerce live video displays of goods provide more complete, accurate, and efficient information transfers than traditional online shopping pictures and text displays, which may affect the audience’s product awareness. Contrarily, e-commerce live broadcasts provide users with the opportunity to communicate with the anchor “face-to-face”. Viewers can consider others’ attitudes toward the product and the live broadcast; other participants’ real-time comments, likes, shares, and purchases; and other environmental clues in their own consumption decisions. Generally, e-commerce live streaming effectively delivers intricate product details and environmental cues to viewers by utilizing a unique presentation style and engaging multiple participants, distinguishing it from traditional e-commerce. Consequently, merchants hope to influence consumer perceptions and convince them to participate in live broadcasts and purchase goods through the details provided during a live stream. It is important to not only understand how viewers process different information types in the evolving live commerce landscape, but also to explore this landscape’s influence on viewers’ live shopping behavior, and especially the influence of user-generated content (UGC) on purchase intentions [4]. Bullet screen is a function that allows websites to display real-time comments as subtitle streams in a specific area on a live screen [5]. They resemble overlaying instant comments on top of the live screen. Users engage with the live broadcast by entering the comment stream, sharing, liking, and participating in sweepstakes, thereby contributing to the creation of bullet screens for real-time display. These screens empower viewers to ask questions or share opinions while watching a live shopping broadcast. User-generated bullet screens can pop up in real time on the live broadcast screen, enabling synchronized messages to be seen by other users and anchors. By facilitating communication and connection between content creators and viewers, bullet screens transform the original static and closed live content, providing continuous added value to the content. Bullet screens in live broadcasts reflect audience reactions to product consumption, representing a new form of dynamic and instantaneous information consumption [6]. This emerging social media trend transcends spatial and temporal limitations. Users can express themselves, display their personalities, and receive instant feedback, leading to a sense of satisfaction from collective recognition and a sense of belonging to a group. This strengthens the psychological link between consumers and live broadcasts, deepening emotional connections and community cohesion, thereby influencing product perception. Thus, it presents a new research angle for UGC analysis.

In a live e-commerce context, UGC primarily refers to bullet screens, which are crucial in e-commerce live streaming’s success [7,8,9]; however, their effect on consumer purchase intentions has not been consistently confirmed. Some studies have found that the information quality [10], interaction frequency [11], and a sense of belonging and interactivity [12] among bullet screens can promote consumers’ experiences and online purchase intentions. However, bullet screens may negatively affect user purchase intentions because of the distracting effect [13,14]. Therefore, current research cannot adequately explain bullet screens’ complex effects on live streaming viewers’ consumer behavior. Specifically, the literature must further explore the influence of the information characteristics of bullet screens—such as quality, quantity, emotion, and interaction—on the consumer decision-making process. The elaboration likelihood model (ELM) is often used to study information receivers’ information-processing and attitudinal and behavioral changes in social business situations [15,16]. For example, Gao et al. [17] used the ELM model to explore the audience’s decision-making process through two different routes: the central path (information completeness and accuracy), the edge path (bullet screen consistency and credibility, among other traits), and the peripheral route (bullet screen consistency and trustworthiness, among others) to examine how viewers make decisions. Therefore, the current study addresses which bullet screen information cues influence viewers’ attitudes and shopping decisions in e-commerce live streaming contexts by using the exhaustive likelihood model as a theoretical basis.

The majority of existing studies begin with the short-term effects of live product realization, such as low-priced promotions and Netflix with goods, and ignore the long-term development goal of the unity of product effects, which is not conducive to the brand image of the live broadcast [18]. Therefore, the question of how to improve the long-term brand-building effect with a new means of live broadcast marketing is crucial. Guan et al. [19] suggested that customer eyeballs are easy to blog, attention is second, and the sense of belonging generated by the live broadcast room is rarer. This concept of transcending customer loyalty and embodying the customer’s sense of ownership and connection to the live streaming room at the psychological level is known as the customer’s psychological ownership of the live streaming room. This is an important concept for measuring the relationship between the customer and the live streaming room. This psychological ownership can trigger the “pseudo-endowment effect” to increase customers’ willingness to buy from live broadcasts, as well as promote word-of-mouth and customer fit [20,21,22]. Furthermore, psychological ownership has been shown to play a moderating role in e-shopping. Nguyen et al. [23] revealed that psychological ownership boosts consumers’ perceived benefits of e-shopping, leading to a higher inclination for repurchasing among Generation Z. Even when faced with perceived disadvantages, the presence of psychological ownership can counteract the negative impact and bolster consumers’ confidence in making purchases. Additionally, consumers’ national identity significantly influences their preference for buying local products, with a pivotal role of psychological ownership in this process. Feeling a sense of belonging to a local product enhances consumers’ willingness to make a purchase, especially in environments with strong nationalistic sentiments [24]. Therefore, enhancing customers’ psychological ownership is important for e-commerce live streaming to gain a further competitive advantage. In addition, parasitic relationships in live-streaming platforms, a heavily researched topic, may impact users’ purchase intentions [25]. The parasocial relationship is a unilateral relationship between an audience and a mediator [26], an imagined interpersonal relationship that develops based on a long-term emotional attachment that the audience grows to the mediated figure [27]. Parasocial relationships may contribute to willingness to buy in the live room [28]. Research indicates that the affordability of live-streaming technology helps develop parasocial relationships among viewers, impacting psychological, social, and technological aspects [29]. According to Scheibe et al. [28], social behavior on live streaming services is close to parasocial relationships due to the high level of interactive communication between viewers and live streaming companies/viewers and viewers. Since friendship, self-representation, and understanding are the three components of prosocial relationships [30], wishful identification and contact with other people/anchors in the live stream can develop into PSR [31]. Whereas immediacy, interactivity, and participation related to communicative presence enhance individuals’ friendship, understanding, and identification with other people/anchors in the live room, a robust communicative presence tends to lead to stronger parasocial relationships for individuals. Meanwhile, since social and personal identity is closely related to psychological ownership [32,33], consumers’ understanding and identifiability from live streaming are inseparable from psychological ownership. Thus, parasocial relationships may contribute to users’ psychological ownership of the live stream.

Customers’ sense of presence in a brand’s live broadcast can establish an immersive and intimate shopping atmosphere, reducing the psychological distance between customers and the brand. This, in turn, helps foster a stronger bond between customers and the brand. The sense of communication presence within the broadcasting room signifies a comprehensive and tangible interpersonal interaction that customers experience, highlighting the immediate nature of communication in live broadcasts. Hence, enriching users’ communication presence is vital for enhancing user engagement and boosting sales conversion. Numerous studies have explored the moderating influence of social presence in e-commerce settings, primarily focusing on consumer trust, perceived value, and purchase intention. They reveal that social interaction features in e-commerce can engage potential consumers effectively. Social presence significantly moderates the impact of virtual friendship, emotional investment, and platform attachment on purchase intention [34]. Additionally, UGC quality can instill trust among members in live broadcasting rooms, while communication presence positively moderates the association between trust and purchase intention [35]. Ma et al. [36] categorized social presence into consumer–anchor interaction and consumer–consumer interaction (communication presence) regarding their effects on perceived value and purchase intention. They found that communication presence boosts the correlation between perceived value and purchase intention. It also positively moderates the association between e-service quality and customer loyalty intention in the context of multichannel retailing [37].

Consumer behavior research is increasingly focusing on the mediating role of communicative presence in various contexts, particularly its impact on consumer purchase intention, brand attitude, and loyalty. The social presence of other viewers in a live broadcast, known as communicative presence, directly influences consumer purchase intention. This feeling of presence can support purchase decisions by improving consumer perceptions and trust in the product [38,39]. Moreover, it can boost brand loyalty by enhancing positive attitudes toward the brand [40], triggering emotional arousal [41], and increasing brand engagement [42]. However, the impact of communication presence on purchase intention remains inconclusive. A study indicated that concurrent online presence of viewers did not necessarily result in a feeling of companionship during live shopping [43]. While the immediacy and interactivity of bullet screens could be linked to communicative presence, the influence of UGC on it in live e-commerce settings has not been thoroughly investigated. Thus, it is essential to delve deeper into the mediating role of communicative presence between UGC and purchase intention in live streaming marketing. In summary, this study intends to combine live information cues with the help of an ELM, build a research model for the purchase intentions of users in a live streaming room, and analyze the effects of various kinds of information cues in a live streaming room on shopping behavior. In doing so, it also aims to verify the mediating roles of psychological ownership and communicative proximity in e-commerce live streaming, which have not been thoroughly explored. This research provides specific theoretical references for research on e-commerce live streaming and offers practical guidance for e-commerce live sellers to improve their live marketing strategies.

## 2. Literature Review

### 2.1. Elaboration Likelihood Model

The ELM is a dual-pathway theory regarding user attitude formation and modification. The model indicates that changes in individual attitudes may be caused by two paths: the central and edge paths. The central path factor connects to information content. People who engage in central processing are likely to carefully consider relevant information, analyze arguments, and make thoughtful judgments [44]. Those who evaluate information through the peripheral route place less emphasis on information quality and rely heavily on the source’s credibility and the information environment’s attributes to assess the target’s reliability [45]. E-commerce has extensively employed the ELM to verify users’ opinions or assess reviews posted on the Internet [22]. Live e-commerce innovatively transforms the e-commerce environment into a social, hedonic, and customer-centered blend of video content and live communication and consumption [46], and its messaging has a level of intricacy not found in traditional e-commerce. Consequently, the model must be adjusted and expanded in different situations.

#### 2.1.1. UGC Quality

The UGC quality is defined as the review material’s authenticity and dependability, the content’s significance relative to the product being reviewed, and whether it offers substantial timely and valuable information to future buyers [47]. The UGC quality is crucial for influencing consumer decision making [48], and is the truest reflection of the experience of using products recommended in a live stream. Subjective and empty bullet screens do not provide factual information but only suggestions, including low-quality comments [48]. Furthermore, Lyu et al. [49] discovered that the UGC quality directly and positively affects consumers’ purchase intentions by modeling the structural equations of UGC quality, perceived interactions, and purchase intention in Internet retailing. As consumers will place effort into collecting relevant bullet screen information and judging the bullet screen’s quality only when they have a strong demand for the product or live broadcast, a process that requires a certain level of information processing ability [50], the UGC quality is a central path factor.

#### 2.1.2. UGC Emotion

The UGC emotion refers to the emotional reaction individuals create after browsing bullet screens and given their past experiences and emotional perceptions or judgments, such as the degree of fondness for a product or live broadcast. In e-commerce live streaming situations, the emotional tendencies expressed on the bullet screen are crucial for creating a positive live streaming atmosphere, and positive emotional bullet screens promote consumers’ charitable purchasing behaviors, such as the purchase of help products [51]. Negative emotional bullet screens increase consumers’ risk perceptions, and trust and perceived risk have a mutually exclusive connection [52]; thus, negative information reduces consumers’ trust in live streaming. Some scholars have also analyzed various online comments’ effects on purchase intentions, as stated by the intensity of emotions [53]. For example, Meng et al. [54] constructed a path equation regarding the influence of emotions and online purchases in live streaming rooms and observed that exuberance can increase the search and purchase of products recommended in live streaming rooms. Additionally, external information content is intricately linked to prospective consumers’ choice of paths; the center path is activated when consumers engage in the cognitive processing of information, and the edge path is activated when consumers process emotional information [55]. In e-commerce live streaming contexts, other viewers’ bullet screens reflect their perceptions and emotions of live streaming content to a certain extent, providing consumers with heuristic cues to evaluate products. Therefore, this study considers UGC emotions as a key edge cue representing the emotional tendencies expressed in the bullet screen.

#### 2.1.3. UGC Interaction

The interaction of the network is essentially a type of social interaction [56], or, specifically, the process of spreading information and interdependent behavior between individuals or groups through language and expressions, among other factors [57]. The UGC interaction manifests in the exchange of information that helps users solve problems or participate in live interactions, such as liking posts, sharing products, or live streaming, which are important means for consumers to search for information online [58]. Users can share and exchange details related to live streaming products and services with others on a live streaming platform [59]. This removes uncertainty and makes the bullet screen information more reliable [60]. Retweets and likes on bullet screens can suggest degrees of consumer empathy, with higher empathy increasing consumers’ recognition of the value of a product or live stream [61]. Previous research highlighted that brand engagement, including UGC interactions, positively affects loyalty purchase intentions [62]. Individuals process information through the edge path when they strongly emphasize the trustworthiness of the information source and the context in which the information is provided [44]. Therefore, UGC interactions are part of the edge path precisely because UGC interactions reduce the risk and uncertainty in purchasing decisions through sharing, liking, and sweepstakes [63] and increase credibility.

### 2.2. Purchase Intention

This study denotes purchase intention as the degree to which consumers are ready to buy a product through live streaming [64,65]. As this is a key predictor of purchasing behavior, a further exploration of purchase intentions is necessary for a deeper understanding of live streaming purchasing behavior. Purchase intentions relate to various factors, including UGC quality [66] and interaction [67], which positively impact purchase intentions. Positive perceptions of online UGC can also promote consumers’ willingness to purchase such content [62]. Additionally, the literature has also demonstrated the influence of factors such as the culture of the social network [68], perceived usefulness and perceived pleasure [69], and psychological distance and perceived uncertainty [70] in live streaming environments on the willingness to buy.

### 2.3. User-Generated Content (UGC)

UGC was first proposed by Meeker in the 2005 China Internet Industry Report, and broadly refers to the text, images, audio, video, and other content that users post on social platforms [71]. Bullet screens are carriers of UGC in live sales activities [72]; this can be interpreted as a unique form of UGC, which is defined by large-scale interactions and screen-centeredness that occurs in real time [73]. It has also been observed that discovering potential information from UGC, such as bullet screens, helps improve recommendation performance [74]. Therefore, this study defines UGC as the text, emojis, and streams of captions sent by consumers during live broadcasts because of such interactive behaviors as sharing, liking, and lucky draws. In the virtual online shopping environment, bullet screens’ UGC has become an important innovation resource and is at the core of e-commerce platforms’ competitiveness. From one perspective, UGC is conducive to live viewers’ mastery of product details and communication through personal recommendations [75]. However, bullet screens reflect the merchant’s credibility, which can reduce consumers’ risk and uncertainty regarding the standard of the product or service before purchasing, and eliminate information asymmetry. In summary, UGC may have a beneficial effect on purchase intentions.

UGC’s impact on consumption intentions has been a focus in related fields [76,77,78], and existing studies have cultivated two research trends at the objective and subjective levels. The former identifies UGC’s influence on purchase intentions through quantitative attributes, such as the number of reviews [79], ratings [80], length of reviews [81], and review quality [49]. The latter explores consumer attitudes and behaviors in subjective emotional situations [54]. Bullet screens sent by consumers during live broadcasts often contain emotions that potential consumers can perceive differently. However, the UGC has multiple paths of influence on purchase intentions that are frequently caused by the interplay of various factors. Therefore, this study combines the subjective and objective features of UGC to elucidate user purchase intentions during live broadcasts.

To summarize, this study discusses the impacts of UGC from live streaming bullet screens on purchase intentions in terms of UGC quality, UGC sentiment, and UGC interaction. Therefore, the following hypotheses are proposed:

**Hypothesis 1 (H1a).**  *UGC quality positively impacts viewers’ purchase intentions in e-commerce live rooms.*

**Hypothesis 1 (H1b).**  *UGC emotion positively impacts viewers’ purchase intentions in e-commerce live rooms.*

**Hypothesis 1 (H1c).**  *UGC interaction positively impacts viewers’ purchase intentions in e-commerce live rooms.*

### 2.4. Psychological Ownership

Psychological ownership is the mental state in which a person views something as belonging to them, a kind of perceived possession of a target [82]. It is fundamentally a psychological bond between the buyer and the objective, emphasizing the consumer’s sense of ownership of the objective relative to consumption [83] with cognitive and affective components [84]. Individuals in this psychological state often treat the target possession as a continuation of their own being or an expression of their self-concept [82]. Control, commitment, and the deep understanding of a target can contribute to individuals’ strong sense of ownership [82]. More substantial contact behaviors with a product, such as customer engagement, interaction, and the adoption of new technologies, can influence customers’ psychological ownership by increasing their sense of control [85]. Selling behavior in live streaming rooms is a newer form of selling compared to the traditional offline model. Consumer behaviors, such as sending bullet screens, liking, and sharing in a live streaming room, are all manifestations of their participation and interaction. All these behaviors can increase consumers’ sense of control over the live stream and its products, which then increases their psychological ownership. Therefore, UGC interactions may positively affect psychological ownership. Current research on customers’ psychological ownership involves two levels: customers’ psychological ownership of brands (collective) and products (individual) [85,86]. Previous studies have mostly explored the individual perspective, although collective psychological ownership is also important. In collectivist cultures, such as China, individuals are more likely to generate collective than individual psychological ownership [87], and collective psychological ownership must still be further explored [88]. Therefore, the current research focuses on consumers’ collective psychological ownership of the Oriental Selection live streaming room on Douyin.

In summary, this study formulates various hypotheses. Once a consumer establishes psychological ownership, he or she will attempt to acquire formal ownership to enhance their sense of control and disposition toward a product. It has been demonstrated that the greater the consumer’s psychological ownership, the greater their degree of preference for possession and the sense of mental rejection of other options, as well as the stronger the control motive and desire for possession [89,90]. Additionally, the theory of the endowment effect reveals that psychological ownership leads to a greater perceived value of possessions and fosters an emotional attachment to them. Alternatively, it increases the sensitivity and degree of pain in the loss of a possession [91], therefore enhancing the willingness to buy. In summary, this study proposes the following hypotheses:

**Hypothesis 2.**  *Psychological ownership positively influences viewers’ purchase intentions in e-commerce live rooms.*

**Hypothesis 3 (H3a).**  *UGC quality positively influences viewers’ psychological ownership in e-commerce live rooms.*

**Hypothesis 3 (H3b).**  *UGC emotion positively influences viewers’ psychological ownership in e-commerce live rooms.*

**Hypothesis 3 (H3c).**  *UGC interaction positively influences viewers’ psychological ownership in e-commerce live rooms.*

### 2.5. Communication Presence

Communication presence is the extent to which a person perceives the effectiveness of instantaneous communication with others in live interactions [92]. It is one of the primary factors of the three-dimensional model of social presence [39], which is the embodiment of the advanced real-time and three-dimensional interaction characteristics of social presence within the webcasting field. Communication presence contemplates the immediacy and three-dimensionality of customers interacting and communicating with others on the live broadcast platform. This differs from the previous online web shopping context of presence (emphasizing the degree of physically experiencing others); it reflects the full and in-depth controllable interpersonal interactions that customers obtain in the live room, and better mirrors the live room’s instantaneous communication characteristics. Customers not only exist in a common virtual space with others in the living room, but also communicate through real-time language and text to send bullet screens, likes, shares, and other rich forms of interaction. In doing so, people are more prone to experiencing a feeling of communicative presence with others than with traditional web pages. Therefore, this study introduces communication presence as a scene characteristic of live streaming rooms. Instant UGC (bullet screens) on the live streaming platform mitigates any emotional separation between consumers and the live streaming room as well as other customers, enhances the fluidity of real-time communication between individuals, restores the interactive scene during offline shopping, and enhances an individual’s sense of immersion in their consumption. Gong [93] noted that real-time interactions and consumer-to-consumer feedback in the live room are important methods to increase the sense of presence; they are also critical for improving consumers’ trust in the online platform. As UGC is the main form of individual interaction and communication in a live room, the level of UGC increases the sense of communication presence. The more pronounced the feeling of communication presence among consumers, the higher their sense of identity, trust, brand commitment, and consumption intentions [42,94,95]. Therefore, this study proposes the following hypotheses:

**Hypothesis 4.**  *Communication presence positively influences viewers’ purchase intentions in e-commerce live rooms.*

**Hypothesis 5 (H5a).**  *UGC quality positively influences viewers’ communication presence in e-commerce live rooms.*

**Hypothesis 5 (H5b).**  *UGC emotion positively influences viewers’ communication presence in e-commerce live rooms.*

**Hypothesis 5 (H5c).**  *UGC interaction positively influences viewers’ communication presence in e-commerce live rooms.*

**Figure 1:**
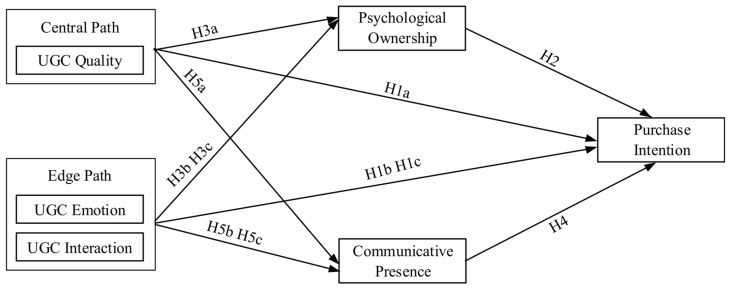
Research model.

Building on the previously discussed theoretical framework and the research hypotheses, this study proposes the following theoretical framework, as depicted in Figure 1. In this framework, the UGC quality is a center path factor, and UGC emotion and UGC interaction are edge path factors. Psychological ownership and communication proximity are mediating variables, and the dependent variable is the consumer’s willingness to purchase the item showcased in the live room. This study uses the fine processing theory as its theoretical basis and UGC as the independent variable to examine the conduction mechanism of consumers’ intentions to purchase during e-commerce live streaming.

## 3. Materials and Methods

### 3.1. Questionnaire and Measurements

This study primarily used the Questionnaire Star Platform for questionnaire distribution. Table 1 lists the questionnaire’s specifics.

To collect users who are actually using the Oriental Selection Live Streaming Room on Douyin, it offers a diverse range of products, including clothing, cosmetics, electronics, home appliances, fresh food, and snacks. The questionnaire was set with restrictive questions. The first question was set as “Have you watched the sales live broadcast of the Oriental Selection Live Streaming Room on Douyin?” The questionnaire would be terminated in advance if the answer were negative. 

The questionnaire link was clicked 492 times, and 404 copies were obtained by removing invalid questionnaires, with an efficient recovery rate of 82.08%. The research participants’ personal characteristics include their gender, age, highest education, marital status, and monthly income. To examine the mechanism regarding UGC’s influence on purchase intentions in the Oriental Selection live room on Douyin, the survey used a five-point Likert scale, with responses ranging from one (“completely disagree”) to five (“completely agree”) for the five latent variables: UGC quality, UGC emotion, UGC interaction, communication proximity, and psychological ownership. 

### 3.2. Sample Description

This study focuses on examining how UGC in live e-commerce broadcasting influences purchase intention and its underlying mechanism. Taking Oriental Selection on Douyin as a case study, we categorized UGC into three dimensions: quality, emotion, and interaction. Employing the elaboration likelihood model (ELM), we introduced psychological ownership and the sense of communicative proximity to investigate their role as mediators in this process.

The selection criteria for the sample were users who viewed the live sales broadcast in the Oriental Selection Live Room on Douyin. Based on statistical results, the study’s research population was primarily composed of employed women, aged between 26 and 30, holding undergraduate degrees, with a typical monthly salary ranging from CNY 5001 to 10,000. The sample’s characteristics aligned closely with the general profile of the platform’s consumer base. Table 2 presents the descriptive statistics of the survey participants’ demographic factors.

The research revealed that, regarding gender, females made up a greater part of the overall population than males, or nearly twice as many, at 60.15%, which could be linked to women’s heightened inclination toward consumption. Regarding age, the primary research team was the 26–30 age group, followed by individuals aged 18–25, both of whom cumulatively accounted for 55.94%. This indicates that the younger group had more purchasing power and a stronger willingness to consume. In terms of occupation, 42.82% worked in enterprises and institutions, followed by 34.9% self-employed people and freelancers. Other occupations accounted for the lowest proportion, with only eight people. In terms of educational background, graduate students and above accounted for the smallest proportion at only 15.35%, and 50.50% had undergraduate degrees as the largest group. In terms of average monthly income, those with CNY 5001 to 10,000 accounted for the largest proportion, or approximately one-third overall, followed by those with CNY 3001 to 5000, at 28.22%. The smallest percentage included those with CNY 10,000 or above, at only 16.83%.

### 3.3. Statistical Analysis

The collected data underwent reliability and validity tests, descriptive statistics, and correlation analysis using SPSS 27.0. Reliability was assessed by Cronbach’s alpha, while structural validity was determined through exploratory factor analysis. Demographic characteristics were depicted using descriptive statistics like frequencies and percentages. Correlation analysis gauged the relationships between variables. Subsequently, structural equation modeling with AMOS 25.0 illustrated the interaction pathways between variables. This multivariate method, commonly employed in the social and behavioral sciences, enabled hypothesis testing and modeling in this study [107]. The model fit index compared the hypothesized and observed data, followed by standardized path analysis to confirm proposed hypotheses.

## 4. Results

### 4.1. Reliability Analysis

A reliability analysis was employed to compare the consistency and dependability of the responses to quantitative data, particularly concerning questions related to attitude scales. The alpha coefficient was then calculated. A value exceeding 0.8 suggests high reliability; a value falling between 0.7 and 0.8 suggests strong reliability; an acceptable level of reliability is suggested if the value falls between 0.6 and 0.7; and an indication of poor reliability is given if the value is lower than 0.6 [108].

Table 3 presents the results of reliability analysis.

According to Table 3, the reliability coefficient values of UGC quality, UGC emotion, UGC interaction, communication proximity, psychological ownership, and the willingness to purchase all ranged from 0.80 to 0.99. As these values are greater than 0.8, this demonstrates that each variable in the questionnaire possesses high reliability. The questionnaire’s total reliability was 0.942, which exceeded 0.8, indicating excellent reliability. In conclusion, the research data exhibited high reliability.

### 4.2. Validity Analysis

A factor analysis was used to determine validity. Table 4 presents the results.

Table 5 presents the total variance explained.

Factor-loading coefficients were used to measure the factor (dimension) and question item correspondence. An orthogonal rotation of the factors by the maximum variance method was conducted to acquire a table of the rotated factor-loading coefficients. Table 6 displays the outcome.

First, the KMO value was examined to determine the research data’s suitability for information extraction. A KMO value greater than 0.8 indicates high suitability, while a value between 0.7 and 0.8 suggests good suitability. A value between 0.6 and 0.7 indicates moderate suitability, and a value below 0.6 denotes unsuitability for extracting information. If the KMO exceeds 0.6, this would signify a correlation among the variables, meeting the prerequisites for a factor analysis. A significance level of *p* < 0.05, as determined by Bartlett’s test, confirms the feasibility of conducting a factor analysis [109]. 

Second, the correspondence between the items and factors was examined. If the correlation aligns with the study’s psychological hypotheses, then the validity is considered high; in cases of low validity, the correspondence is grossly inconsistent with expectations, or an analytic item corresponds to a covariance value of less than 0.4. In that case, the item may be eligible for removal. Furthermore, standardized criteria exist for removing items. One is that the commonness value is less than 0.4; the other is that a serious deviation occurs among the analyzed item and factor correspondences. The previous four steps were then repeated until the KMO reached the standard. The question item and factor correspondences essentially parallel expectations, which ultimately indicates good validity [110]. As Table 4 demonstrates, the KMO measure is 0.945; Bartlett’s test of sphericity (*p* < 0.001) suggests that the variables correlate, and that the study data are appropriate for a factor analysis to extract useful information.

A principal component analysis was used to conduct the exploratory factor analysis, and Table 5 presents the total variance explained. This table indicates that six male factors were analyzed following the rotation, and all their eigenroots exceeded 1; these factors’ explained variance values were 18.248%, 14.221%, 9.765%, 9.743%, 9.572%, and 7.582%, respectively. The total contribution of the questionnaire variables’ explanation amounted to 69.131%. As this was greater than 60%, the questionnaire items exhibited a strong explanatory power for the variables. Hence, it is possible to effectively extract information from these research items.

Table 6 indicates that Factor 1 is associated with the communication proximity variable because the factor loadings of each communication proximity topic are greater than 0.5 on Factor 1. Similarly, Factors 2, 3, 4, 5, and 6 are associated with UGC interaction, UGC emotion, UGC quality, psychological ownership, and purchase intentions, respectively. Commonality values were used to exclude unrealistic research topics. This suggests that all common factors had variances exceeding 0.4, indicating that the information related to the research items could be accurately extracted. In conclusion, the questions’ alignment with the variables remained consistent with the questionnaire design’s assumptions, demonstrating that the questionnaire possesses strong structural validity.

### 4.3. Correlation Analysis

Table 7 presents the results from a Pearson’s correlation analysis of UGC quality, UGC emotion, UGC interaction, communication proximity, psychological ownership, and purchase intention. The analysis reveals a significant, positive correlation between UGC quality and the purchase intention (*r* = 0.467, *p* < 0.01), between UGC emotion and purchase intention (*r* = 0.460, *p* < 0.01), between the homogeneity of UGC interactions and purchase intention (*r* = 0.476, *p* < 0.01), and between communication proximity and the purchase intention (*r* = 0.449, *p* < 0.01). Furthermore, a strong positive correlation exists between psychological ownership and the purchase intention (*r* = 0.480, *p* < 0.01). Psychological ownership has the strongest positive correlation with purchase intention, followed by UGC interaction, UGC quality, UGC emotion, and communication proximity.

### 4.4. Structural Equation Modeling

As an applied analytical approach, structural equation modeling (SEM) is a further evolution of regression and path analyses. It is more suitable for the study of complex causality, as it has the advantage of handling several dependent variables simultaneously and allowing greater flexibility in the measurement model. In this study, SEM was used to obtain the follow-up test results.

#### 4.4.1. Model Fit Test

This study analyzed the goodness of fit of the research model formed by UGC quality, UGC emotion, UGC interaction, communication proximity, psychological ownership, and purchase intention. Table 8 displays the results and Table 9 lists the factor-loading coefficients.

Table 8 displays three rows presenting the fitting index, statistical judgment criteria, and the fitting value of the research model. All indicators are within the normal range, suggesting that the research model’s fit is more suitable and warrants further analysis [111]. The factor-loading coefficients were screened for the measurement variables within the factor, as well as the measurement variables, and all passed the significance test (*p* < 0.05). The standardized loading coefficient value was greater than 0.4, suggesting that the measured variables satisfied the factor criteria; otherwise, the variables would be considered for deletion [112]. Table 9 lists the factor-loading coefficients. The information in this table demonstrates that the individual topics of specialization are statistically significant (*p* < 0.001), while all their standardized loading coefficients are greater than 0.4, which can be deemed to exhibit a sufficient explained variance to allow the items to be presented using identical elements. Similarly, the individual questions for each of the other variables are clearly significant, with standardized loading coefficients greater than 0.4, indicating that the individual variables strongly correlate.

#### 4.4.2. Main Effect Test

**Figure 2:**
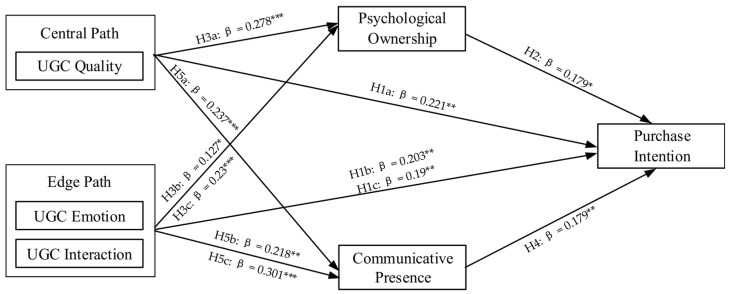
Structural equation model results. Note: *** *p* < 0.001; ** *p* < 0.01; * *p* < 0.05.

In SEM, the path coefficients indicate the magnitude of the direct associations between the model’s variables. After establishing the structure’s reliability and validity, we evaluated the SEM. Figure 2 illustrates the SEM results, specifically. 

Table 10 demonstrates that most of the hypotheses are confirmed based on the data.

The results demonstrate that UGC quality (β = 0.221, *p* < 0.01), UGC emotion (β = 0.203, *p* < 0.01), and UGC interaction (β = 0.19, *p* < 0.01) are strong predictors of e-commerce live streaming purchase intentions regarding the factors that influence purchase intentions from both the center and edge path cue perspectives. As we hypothesized that UGC quality, UGC emotion, and UGC interaction would positively impact purchase intentions in e-commerce live rooms, H1a, H1b, and H1c are supported.

In terms of the effects of the variables of psychological ownership and communicative proximity on purchase intentions, psychological ownership (β = 0.179, *p* < 0.05) and communicative proximity (β = 0.179, *p* < 0.01) were antecedents of purchase intention. This suggests that psychological ownership and communicative presence have a beneficial impact on the willingness to purchase in a live room; these results support H2 and H4. Among the influences on psychological ownership, UGC quality (β = 0.278, *p* < 0.001) and UGC interaction (β = 0.23, *p* < 0.001) had a notable beneficial impact on psychological ownership, and UGC emotion (β = 0.127, *p* = 0.059) had a borderline notable beneficial impact on psychological ownership. As we hypothesized that UGC quality, UGC emotion, and UGC interaction would positively influence the psychological ownership of e-commerce live streaming, our results support H3a, H3b, and H3c.

In terms of the factors influencing communication presence, UGC quality (β = 0.237, *p* < 0.001), UGC emotion (β = 0.218, *p* < 0.01), and UGC interaction (β = 0.301, *p* < 0.001) had a notable beneficial impact on communication presence. As we hypothesized that UGC quality, UGC emotion, and UGC interaction positively influence communication presence, the results support H5a, H5b, and H5c.

#### 4.4.3. Mediating Effect Test

The hypothesized model suggests that communication proximity mediates the relationship between anthropomorphic cues and live e-commerce purchase intention. The researchers used the bootstrap method suggested by Preacher and Hayes [113] to examine the mediating effect. A sample of 5000 was chosen, with a confidence level of 95%. The confidence intervals for the lower and upper bounds were calculated to test whether the indirect effect was significant. If zero is included in the interval, the indirect effect is not statistically significant. Table 11 indicates that the mediating effects of communication proximity between UGC quality cues, UGC emotion, UGC interaction, and e-commerce live purchase intentions are all significant.

The hypothesized model suggests that psychological ownership mediates the connection between UGC and e-commerce live streaming purchase intention. Researchers used the bootstrap method suggested by Preacher and Hayes [113] to examine the mediating effect. A sample of 5000 was chosen with a 95% confidence level. The confidence intervals for the lower and maximum limits were calculated to test whether the indirect effect was significant. If zero is included in the interval, this suggests that the indirect effect is not statistically significant. The findings in Table 12 indicate that the mediating effects of psychological ownership on UGC quality, UGC sentiment, UGC interaction, and e-commerce live purchase intentions are all significant.

## 5. Discussion and Conclusions

This study considered the ELM to examine the impact of UGC on viewers’ purchase intentions from live streaming with psychological ownership and communication presence as the mediating variables. It also further elucidated the mediating mechanisms of psychological ownership and communication presence through a questionnaire survey, obtaining the following conclusions.

First, this research revealed a beneficial impact of UGC quality on live streaming viewers’ purchase intentions. This aligns with the central path theory in the ELM, implying that information quality is a key determinant of consumer behavior. Specifically, high-quality UGC provides richer and more persuasive information, which helps viewers make informed purchasing decisions. This finding is consistent with those of previous studies. For example, Kim and Johnson [98] observed that the UGC quality significantly influences consumers’ product attitudes and purchase intentions, with information credibility [114], timeliness, and relevance [115] as core influencing factors. Similarly, Mudambi and Schuff’s [116] study found that viewers tend to trust high-quality reviews more when choosing to purchase highly complex products. Additionally, Filieri and McLeay [117] noted that the accuracy of information, and especially its usefulness, is the main factor affecting its importance in consumers’ decision-making processes. However, the results of this study are inconsistent with those of other studies. For example, Ghose and Ipeirotis [118] noted that although high-quality reviews attract consumers’ attention, their direct effect on the final purchase decision is not as significant as that of other factors (e.g., price sensitivity and brand effects). This may indicate differences in the UGC’s influence and mechanism of action in different purchase environments and product types. By applying the theory of fine processing possibilities to live streaming in an e-commerce environment, this study investigates how interactivity and immediate feedback unique to live streaming can enhance the impact of UGC quality. This contributes to the current body of e-commerce knowledge while providing guidance for practical maneuvers, suggesting that live streaming sellers can improve the quality and relevance of UGC to enhance consumers’ purchase intentions. Furthermore, these research results suggest a direction for future studies to delve deeper into the specific mechanisms by which different types of UGC (e.g., video content and real-time interactions) influence consumer behavior. These studies are important for understanding and utilizing live streaming platforms’ commercial potential.

Second, UGC emotions and UGC interactions significantly and positively affect the buying intentions of users watching live streams. Emotionally enriched UGC can trigger consumers’ emotional resonance; according to the theory of affective transfer [119], positive emotional expressions can enhance consumers’ positive emotional experience, thus increasing purchase intentions. For example, Luo [120] demonstrated that emotional content is more likely to elicit empathy from viewers, which is particularly evident in real-time interactions with live streaming platforms, while the UGC’s interactivity—and especially bullet screen interactions between viewers and between anchors and viewers—enhances the trust in and persuasiveness of the message, according to social influence theory [121]. Moreover, Sun et al. [122] also confirmed that interactive UGC can enhance consumer trust in anchors and goods, which then promotes purchasing behavior. However, this study’s results also reveal some inconsistencies compared to previous ideas in the literature. For example, Berger and Milkman [123] stated that while emotional content can increase information dissemination, it does not always directly influence consumption decisions. Tang et al. [124] found that excessive emotionality may lead to low information credibility and negatively affect purchase intentions.

These results present a certain complexity given the edge path from the ELM, which suggests that in addition to the central path (i.e., a cognitive process involving the thoughtful consideration of information), an edge path exists (i.e., the surface processing of information). The edge path’s role may be worth emphasizing among live streaming viewers, whose purchase intentions are influenced by UGC emotions and interactions. On the one hand, the UGC’s emotional expression and interactivity can directly stimulate the audience’s emotional resonance and engagement, which is closely associated with the emotional processing from the edge path. According to the ELM, the surface processing of emotions may be important in viewers’ formation of attitudes and decision making. Therefore, when UGC emotions and interactivity are high, viewers may be more inclined to evaluate products or brands through emotional resonance and emotional engagement rather than thoughtfully considering the credibility and relevance of their information. On the other hand, although the ELM model emphasizes the central path’s importance in cognitive processing, the edge path’s emotional processing may also indirectly affect the central path’s cognitive processing. For example, an UGC with high emotional expression and interactivity may trigger viewers’ emotional engagement, which enhances attention and memory of information; in turn, this affects cognition and attitude formation toward the product or brand. However, it should be noted that the edge path’s emotional processing may have certain limitations. The ELM indicates that the edge path’s effects are usually more transient and superficial, and not as long-lasting or as in-depth as those of the center path. Therefore, although UGC emotions and interactions can directly influence viewers’ purchase intentions, their long-term effects may need to be supported and solidified by cognitive processing in the center path. The findings can be better understood through the lens of the parasocial relationship theory (PSR). It refers to a connection that goes beyond boundaries, exemplifying the bond between social media users and influencers [125]. It involves more than just observing and interacting with influencers online; rather, it encompasses the development of intimate relationships through interactions in an online media platform [30]. Three dimensions of PSR were utilized in this study: friendship, self-representation, and understanding. It is often noted that users share their thoughts, emotions, and attitudes on the live stream, forming equal and reciprocal friendships with other consumers [126]. They also strive to understand the personal cognitive feelings conveyed in the bullet screens [127], thus establishing a parasocial relationship. The UGC quality and interactions enhance the sender’s relationship with the audience, fostering parasocial connections. Users’ perception of influencers on social media correlates with heightened positive emotions [128], closely tied to these parasocial bonds. Various research exhibited the affirmative influence of such relationships with media influencers on buying intentions [98,129,130,131,132,133,134]. Engaging and sharing experiences in virtual social spaces can boost consumer trust and identification with a product or service, aiding in purchase decision making [98]. Additionally, parasocial connections formed via sharing and commenting in online communities significantly boost the willingness to buy recommended products. This mechanism primarily relies on emotional resonance and social validation, encouraging consumers to purchase based on recommendations from social networks [132]. Furthermore, the communicative presence moderates the impact of UGC (quality, emotion, and interaction) on purchase intentions. The mediating role of communicative presence is critical in e-commerce environments, and particularly in the connection between UGC and purchase intentions. Participants from collectivist cultural backgrounds will pay more attention to situations [135]. The communicative presence describes the degree of immersion and real-time interaction users experience in an interactive environment. This psychological state mediates the UGC’s enhancing and influencing of users’ purchase decisions. The quality, emotional richness, and interactivity of UGC directly shape users’ communication presence. High-quality content provides rich and accurate product information, helping users establish preliminary knowledge of the product. Emotional content can evoke users’ emotions and cause them to emotionally resonate with the content. In contrast, interactivity improves user engagement through participation and feedback mechanisms, making the user’s experience in the virtual environment more realistic and attractive. These UGC features enhance the user’s communication presence, or the real-time nature and effectiveness of the communication and interaction the user experiences. Simultaneously, when users are highly present during the interaction process, their perceptions and attitudes toward the product are more likely to transform into actual purchasing behaviors. This is because a sense of presence enhances the user’s trust in and satisfaction with the virtual environment, making the virtual experience close to or equivalent to a real experience. Therefore, communicative presence as a mediator effectively transforms the positive qualities of UGC and enhances purchase intentions. Previous research has indicated that the reinforcement of presence can significantly enhance users’ propensity to act; for example, Huang and Benyoucef [136] found that presence can significantly enhance purchase intentions in social commerce environments. Similarly, Skadberg and Kimmel [137] observed that increased presence directly increases users’ willingness to book online travel services. Therefore, understanding and optimizing the communication presence as a mediator between UGC and purchase intentions is key to improving conversion rates among e-commerce platforms. Such platforms should enhance the user’s sense of presence by increasing the content’s interactivity and emotionality as well as the user-friendliness of the interface and real-time feedback mechanisms to effectively encourage purchasing behavior. Understanding and applying this mediating role can help e-commerce platforms design more effective user-interaction strategies and content-distribution plans to enhance users’ satisfaction and loyalty.

Finally, psychological ownership mediates the effect of UGC (quality, emotion, and interaction) on purchase intentions. It pertains to the psychological condition in which a person interprets the object as owned by him or her, and can shape the consumer’s feeling of control and intimacy with the item through UGC, thus enhancing purchase intentions. UGC influences consumers’ psychological ownership through quality, emotions, and interactive characteristics. High-quality UGC provides comprehensive, authentic product information, enabling consumers to enhance their comprehension of a product’s characteristics and advantages, thus creating a strong sense of “ownership”. Emotionally charged content can evoke emotional resonance in consumers, connecting their state of mind to that of the product. Additionally, the interactive nature of UGC—including liking, commenting, and sharing—provides consumers with a sense of involvement and makes them feel that they can influence a product’s development or significantly impact a community, enhancing their psychological ownership of the product. Psychological ownership also significantly affects consumers’ willingness to buy. When consumers experience the psychological ownership of a product, they are more inclined to remain connected to it to avoid losing their possession. Therefore, psychological ownership drives consumers to develop a preference for currently owned products in the face of competing products, creating a unique type of exclusivity. This also leads to higher emotional attachment, which increases consumers’ willingness to purchase a product or devote more resources to it. Heightened psychological ownership results in consumers’ more positive evaluations of a product, thereby increasing its perceived value. 

In summary, psychological ownership is an important mediator, as it transforms the positive qualities of UGC into enhanced purchase intentions. It establishes consumers’ emotional connections with and sense of control over the product through UGC, which enhances their perceived worth of the product and facilitates their purchase. Therefore, platforms should enhance their UGC quality, emotionality, and interactivity to increase consumers’ psychological ownership, which will ultimately translate into the realization of actual purchasing behavior. This understanding can help platforms and brands design content strategies that enhance consumers’ psychological ownership and improve marketing effectiveness.

## 6. Research Implications

### 6.1. Theoretical Implications

This research aimed to investigate the effect of UGC on purchase intention in an Oriental Selection live streaming room on Douyin, and especially the mediating roles of psychological ownership and communicative proximity. This research broadens the use of psychological ownership and communicative presence within e-commerce while offering new perspectives for understanding viewer behavior in e-commerce live streaming rooms. Traditional e-commerce research has widely explored psychological ownership theory. However, its role and impact on the live streaming e-commerce environment have not been fully investigated. The UGC in live streaming, such as viewer comments and anchor interactions, provides rich socialized information and emotional experiences that are key in enhancing consumers’ psychological ownership. Meanwhile, the sense of presence, as the degree of “presence” that consumers feel during live communication, may be another important factor influencing their purchase intentions. This presence may strengthen consumers’ trust in the host and the product, thus enhancing purchase motivations. Additionally, by integrating two mediating variables—psychological ownership and communication presence—this study attempted to construct a more comprehensive theoretical model to explain how UGC influences consumers’ purchase decisions through these psychological mechanisms. Establishing this theoretical framework not only helps academics more deeply understand viewers’ behavioral motivations in e-commerce live rooms, but also provides strategic guidance for the industry so that merchants can optimize live content, enhance user engagement, and, ultimately, drive sales conversion. Therefore, this study’s theoretical importance is rooted in its innovative introduction of the concepts of psychological ownership and communication presence in live streaming e-commerce. In doing so, it offers a fresh theoretical perspective and empirical basis for future research and practice. This study enriches and develops the theory of psychological ownership in e-commerce while offering a scientific basis for strategies to evolve content and user-interaction designs among live streaming platforms.

### 6.2. Practical Implications

This study’s focus is the UGC’s influence on purchase intentions in Oriental Selection’s live streaming room on Douyin, with a goal to provide practical guidance in operations strategies for live streaming e-commerce platforms, enhancing the user experience, and facilitating sales results.

First, the study demonstrates a positive impact of UGC quality, emotion, and interaction on purchase intention. The favorable influence of UGC quality on purchase intention suggests that viewers appreciate the authenticity, timeliness, and informational value of live content. For instance, in headline live broadcasts like Oriental Selection, viewers tend to trust the information shared on bullet screens regarding product features and usage experiences. This trust in UGC assists viewers in making well-informed purchase decisions. Therefore, platform operators and brands need to focus on managing the UGC quality. They should guide anchors to prompt viewers to submit professional and reliable pop-up content and continuously monitor and optimize pop-ups to set a positive content standard across live broadcasts. Furthermore, they should address audience inquiries promptly to enhance the timeliness and relevance of bullet screen content, thereby bolstering audience trust. They should implement a robust UGC audit system to promptly detect and address false or illegal pop-up content to uphold the information integrity of live broadcasts. The significant impact of UGC emotion on purchase intent underscores the importance of enhancing audience’s emotional experiences in live e-commerce settings. Positive emotions experienced by viewers during live broadcasts can boost their connection with the content, brand, and products, leading to a higher likelihood of making purchases. It is crucial for platform operators and brands to focus on managing UGC emotions, improving anchors’ emotional expression skills, and steering viewers towards offering positive emotional responses like encouragement and praise, thereby deepening their emotional involvement. Tailoring bullet screen interactive links to suit the emotional preferences of the target audience, such as sharing experiences or expressing admiration, can further enhance emotional resonance, which can positively affect the sales. Furthermore, live broadcast operators should monitor the emotional direction of messages in real time and adjust the content and marketing strategies promptly to align with the audience’s emotional needs. The positive impact of UGC interaction on purchase intentions underscores the significance of audience engagement in live e-commerce settings. Viewers develop a stronger sense of belonging and participation, leading to increased purchase intention when they interact effectively with anchors, brands, and other viewers. To achieve this, live broadcast operators should focus on UGC interaction management and establish a two-way interaction mechanism between anchors and the audience. This can be achieved by creating engaging interactive links, such as Q&A sessions and voting to encourage audience participation. Creating a social atmosphere by implementing features, such as pop-ups for praise, establishing fan groups, and organizing offline meetups, can help enhance the connection among the audience. Moreover, highlighting and rewarding high-quality bullet screen messages by methods like sending gifts or issuing coupons can further boost their enthusiasm for interaction.

Second, psychological ownership is crucial in promoting consumers’ purchase behavior [82]. By examining the UGC’s impact on the psychological ownership of live streaming rooms, this study helps live streaming platforms understand how to enhance viewers’ sense of belonging to their products through content strategies. For example, highly interactive content in an e-commerce live room can deepen viewers’ perceptions of engagement and control, stimulating their purchase intentions [138]. Psychological ownership mediates the impact of UGC quality, emotion, and interaction on purchase intentions. It suggests that viewers perceive the viewing experience as a part of themselves, shaping their attitudes and behaviors towards live content. In e-commerce live streaming, viewers who feel connected to content creation or expression tend to emotionally engage with the content, leading to a higher purchase intention. Therefore, for managers and platform operators, boosting users’ sense of psychological ownership in the viewing experience is crucial for increasing their purchase intent. To accomplish this, platforms can enhance viewers’ engagement and emotional investment in the content through various strategies, for instance, creating a more interactive live broadcast environment, selecting anchors that resonate well with the target audience, and utilizing their influence to boost viewers’ psychological ownership. Additionally, implementing engaging marketing activities, such as limited-time offers and lucky draws, can increase audience participation and strengthen their connection to the live room and brand.

Furthermore, communication presence is another key factor influencing online user behavior, especially in live e-commerce environments [94,139]. This study examined how the communicative presence influences users’ purchase decisions by enhancing their immersion in live content. A live streaming experience with a strong sense of presence promotes trust and builds loyalty [140,141,142], which is important for increasing conversion rates. It has been discovered that communication presence plays a mediating role in the impact of UGC quality, emotion, and interaction on purchase intention. Thus, we suggest boosting audience engagement in live broadcasts by enhancing interactivity and immersion. To achieve this, a variety of interaction options can be offered, such as video links and interactive mini-games, along with bullet screens. Data should be analyzed to identify audience interactive preferences, and customized interactive sessions should be offered to improve engagement. Encouraging audience interaction by providing incentives, like creating a bullet screen interactive ranking, can be adapted. To enhance interaction immersion, it is advisable to improve technical support on the live broadcast platform to ensure smooth and timely interaction. Bullet screen effects, animations, and other visual enhancements in UGC should be utilized to increase audience engagement further. Combining these two psychological mechanisms, this study offers a fresh perspective on the UGC’s significance for live streaming platforms by emphasizing the importance of developing effective content strategies and enhancing user interaction. These insights can help live streaming merchants and platforms design more attractive live streaming scenarios, enhance users’ engagement and purchase intentions, and improve the efficiency and effectiveness of live streaming sales.

## 7. Limitations and Further Research

While this study provides valuable insights into the UGC’s effects on purchase intentions in the Eastern Selection live stream, there are some limitations to consider. First, it employed a design involving a single point in time, which made it difficult to capture temporal changes and causality. Future research could adopt a longitudinal design to track the UGC’s long-term effects on purchase intentions. Furthermore, this research focused on two mediating variables—psychological ownership and communication proximity—however, other psychological mechanisms that may affect purchase intentions were not considered. Future research will focus on investigating advanced mechanistic models in more detail. These models could delve into aspects like the influence of parasocial relationships and different types of products. Furthermore, future studies may also examine the moderating impact of psychological ownership and communication proximity. These efforts aim to develop a more systematic and comprehensive theoretical framework. Additionally, this study’s sample originated from a specific live streaming e-commerce platform, which may include a sample bias that limits the study’s generalizability and replication of the findings. Future studies could broaden the sample size to cover more varieties of live streaming e-commerce platforms to increase the study’s representativeness and credibility. In summary, future research can further improve and expand the content of this study through a longitudinal design and by exploring other psychological variables, expanding the sample’s scope, and following up on the industry’s development dynamics. In doing so, research results can offer more beneficial insights and instructions for theory and practice within the live streaming e-commerce realm.

## Figures and Tables

**Table 1:** Measurement items.

Construct	Item	References
UGC quality (UQ)	UQ1: The bullet screen on the live feed describes the reality of what occurred, and is believable.	[96]
	UQ2: The content provided by the live streaming bullet screen is timely.	[97]
	UQ3: The information contained in the live streaming bullet screen contains valuable tips on featured brands and products.	[98]
	UQ4: The live stream’s bullet screen contains information relevant to that live stream’s content.	[99]
UGC emotion (UM)	UM1: The bullet screens sent by others in the live stream evoke my enthusiasm.	[92]
	UM2: The likes and dislikes reflected in the live streaming bullet screen affect my emotions to a certain extent.	[92]
	UM3: I have some expectations for user-generated content in the live room.	[100]
	UM4: Different emotional atmospheres and emotional intensities of the live streaming bullet screen have different impacts on live streaming sales.	[101]
UGC interaction (UI)	UI1: I received positive responses from the anchor or other viewers regarding the content I posted on that broadcast.	[100]
	UI2: I have participated in the sweepstakes for that live stream.	[100]
	UI3: I retweeted the live feed from that broadcast.	[100]
	UI4: I received likes from other users regarding the content I posted on that live stream.	[100]
	UI5: The live room allows for mutual communication between one or more senders of messages and one or more receivers.	[102]
	UI6: I have seen very little offensive language on that broadcast.	[96]
Communication proximity (CP)	CP1: I experienced a sense of socialization in the live room.	[92]
	CP2: I can consistently engage in conversations with others in the live room.	[92]
	CP3: I can engage in conversations with others in the live room without any problems.	[92]
	CP4: Others could respond to my query on the air.	[92]
	CP5: I have a sense of face-to-face interaction with other members in that live room.	[103]
	CP6: I perceived the personalities of the other members in that broadcast.	[103]
	CP7: I felt the enthusiasm of the other members in that live room.	[103]
	CP8: I experienced what other users felt in that live room.	[103]
Psychological ownership (PO)	PO1: I felt a sense of intimacy watching the live stream in that live room.	[104]
	PO2: I felt like I was in my own live room when watching the live stream.	[105]
	PO3: I felt like the stream/product belonged to me when watching a live stream.	[105]
	PO4: I felt strongly that I could own this live room/product when watching a live stream.	[105]
Purchase intention (PI)	PI1: The user reviews on that live stream enriched my understanding of the products they sell.	[106]
	PI2: The product-related information in the user reviews of that live stream will change my thoughts and attitudes about purchasing.	[106]
	PI3: When buying related products, I will refer to the live user reviews.	[106]

**Table 2:** Respondents’ demographics.

Demographics	Item	Frequency	Percentage (%)
Gender	Male	161	39.85
	Female	243	60.15
Age	18–25	94	23.27
	26–30	132	32.67
	31–40	79	19.55
	41–50	57	14.11
	51–60	35	8.67
	>60	7	1.73
Education	High school and below	138	34.16
	Institutions of higher education	204	50.49
	Postgraduate level or higher	62	15.35
Occupation	Students	82	20.30
	Enterprises and institutions in service	173	42.82
	Self-employed	141	34.90
	Others	8	1.98
Income (CNY)	Under 3000	85	21.04
	3000–5000	114	28.22
	5001–10,000	137	33.91
	Over 10,000	68	16.83

**Table 3:** Reliability analysis results.

Variable	Cronbach’s α
UGC quality	0.853
UGC emotion	0.854
UGC interaction	0.905
Communication proximity	0.922
Psychological ownership	0.859
Purchase intention	0.846
Total questionnaire	0.943

**Table 4:** Results of Kaiser–Meyer–Olkin (KMO) and Bartlett’s tests.

KMO Value		0.945
Bartlett sphericity test	Approx. chi-square	6822.967
	df	406
	*p*-value	<0.001

**Table 5:** Total variance explanation.

CO	Initial Eigenvalue			Extracted Loading Squared Sums			Rotated Loading Squared Sums		
	Total	VP	CU	Total	VP	CU	Total	VP	CU
1	11.246	38.78	38.78	11.246	38.78	38.78	5.291	18.245	18.245
2	2.524	8.702	47.482	2.524	8.702	47.482	4.124	14.222	32.467
3	1.963	6.771	54.252	1.963	6.771	54.252	2.832	9.766	42.233
4	1.609	5.547	59.799	1.609	5.547	59.799	2.826	9.744	51.977
5	1.432	4.936	64.735	1.432	4.936	64.735	2.776	9.573	61.549
6	1.275	4.396	69.131	1.275	4.396	69.131	2.199	7.582	69.131
7	0.589	2.031	71.162						
8	0.56	1.93	73.093						
9	0.529	1.823	74.916						
10	0.514	1.772	76.688						
11	0.498	1.716	78.404						
12	0.468	1.614	80.018						
13	0.441	1.521	81.539						
14	0.436	1.505	83.044						
15	0.415	1.431	84.474						
16	0.404	1.393	85.867						
17	0.39	1.345	87.212						
18	0.377	1.301	88.513						
19	0.363	1.252	89.765						
20	0.347	1.198	90.963						
21	0.343	1.183	92.146						
22	0.334	1.153	93.299						
23	0.323	1.115	94.414						
24	0.307	1.059	95.474						
25	0.3	1.033	96.506						
26	0.284	0.978	97.484						
27	0.258	0.889	98.373						
28	0.254	0.876	99.249						
29	0.218	0.751	100						

Notes: CO = component; VP = variance percentage; CU = cumulative percentage.

**Table 6:** Component matrix and communality (rotated).

Name	Factor Loadings						Communality
	Factor1	Factor2	Factor3	Factor4	Factor5	Factor6	
UQ1	0.165	0.122	0.134	**0.774**	0.165	0.073	0.691
UQ 2	0.136	0.213	0.136	**0.750**	0.179	0.115	0.689
UQ 3	0.185	0.151	0.204	**0.733**	0.168	0.143	0.685
UQ 4	0.158	0.160	0.127	**0.787**	0.089	0.171	0.724
UE 1	0.198	0.199	**0.728**	0.159	0.172	0.125	0.679
UE 2	0.143	0.149	**0.769**	0.132	0.118	0.164	0.692
UE 3	0.140	0.150	**0.784**	0.166	0.135	0.103	0.713
UE 4	0.196	0.173	**0.780**	0.129	0.067	0.098	0.708
UI 1	0.206	**0.717**	0.125	0.178	0.092	0.192	0.649
UI 2	0.153	**0.748**	0.172	0.109	0.224	0.107	0.687
UI 3	0.197	**0.759**	0.166	0.135	0.182	0.037	0.696
UI 4	0.174	**0.790**	0.090	0.058	0.214	0.100	0.722
UI 5	0.179	**0.785**	0.179	0.141	0.068	0.154	0.728
UI 6	0.175	**0.756**	0.091	0.160	0.054	0.115	0.652
CP 1	**0.759**	0.113	0.152	0.136	0.099	0.151	0.663
CP 2	**0.744**	0.180	0.077	0.065	0.143	0.180	0.649
CP 3	**0.781**	0.142	0.085	0.182	0.180	0.101	0.713
CP 4	**0.717**	0.169	0.139	0.075	0.166	0.127	0.612
CP 5	**0.754**	0.129	0.139	0.066	0.146	0.064	0.634
CP 6	**0.769**	0.149	0.092	0.178	0.119	0.106	0.679
CP 7	**0.752**	0.145	0.145	0.126	0.136	0.099	0.651
CP 8	**0.742**	0.197	0.106	0.084	0.145	0.041	0.631
PO 1	0.219	0.215	0.150	0.142	**0.733**	0.137	0.693
PO 2	0.223	0.161	0.110	0.163	**0.768**	0.067	0.709
PO 3	0.266	0.153	0.154	0.136	**0.735**	0.172	0.705
PO 4	0.205	0.188	0.111	0.198	**0.746**	0.146	0.706
PI 1	0.189	0.213	0.183	0.177	0.141	**0.770**	0.759
PI 2	0.223	0.215	0.140	0.167	0.150	**0.777**	0.771
PI 3	0.217	0.155	0.182	0.160	0.196	**0.768**	0.759

Notes: Blue text indicates that the magnitude of the load factor exceeds 0.4. UQ = UGC quality; UE = UGC emotion; UI = UGC interaction; CP = communication proximity; PO = psychological ownership; PI = purchase intention.

**Table 7:** Pearson’s correlation analysis.

	PI	UQ	UE	UI	CP	PO
PI	1					
UQ	0.467 **	1				
UE	0.460 **	0.453 **	1			
UI	0.476 **	0.446 **	0.456 **	1		
CP	0.449 **	0.408 **	0.425 **	0.465 **	1	
PO	0.480 **	0.478 **	0.430 **	0.485 **	0.508 **	1

Notes: ** *p* < 0.01; PI = purchase intention; UQ = UGC quality; UE = UGC emotion; UI = UGC interaction; CP = communication proximity; PO = psychological ownership.

**Table 8:** Model fit test.

Fitting Index	$\chi^{2}$	df	*p*	$\chi^{2}/\mathrm{df}$	GFI	RMSEA	RMR	CFI	NFI	NNFI
Recommended criteria	-	-	>0.05	<3	>0.9	<0.10	<0.05	>0.9	>0.9	>0.9
Fitting value	384.293	362	0.201	1.062	0.945	0.012	0.035	0.997	0.945	0.996

**Table 9:** Factor loading.

Factor	Variable	Coef.	Std. Estimate	*z*	S. E.	*p*
UGC quality	UQ1	1	0.744	-	-	-
	UQ 2	1.048	0.77	14.672	0.071	<0.001
	UQ 3	1.116	0.782	14.883	0.075	<0.001
	UQ 4	1.079	0.781	14.862	0.073	<0.001
UGC emotion	UE 1	1	0.775	-	-	-
	UE 2	0.961	0.759	15.07	0.064	<0.001
	UE 3	1.005	0.776	15.415	0.065	<0.001
	UE 4	0.978	0.771	15.306	0.064	<0.001
UGC interaction	UI 1	1	0.76	-	-	-
	UI 2	1.026	0.791	16.368	0.063	<0.001
	UI 3	1.083	0.795	16.48	0.066	<0.001
	UI 4	1.076	0.798	16.534	0.065	<0.001
	UI 5	1.1	0.815	16.938	0.065	<0.001
	UI 6	0.99	0.745	15.286	0.065	<0.001
Communication proximity	CP 1	1	0.781	-	-	-
	CP 2	0.996	0.768	16.571	0.06	<0.001
	CP 3	1.079	0.821	18.032	0.06	<0.001
	CP 4	0.981	0.746	16.009	0.061	<0.001
	CP 5	0.964	0.75	16.107	0.06	<0.001
	CP 6	1.036	0.792	17.219	0.06	<0.001
	CP 7	1.001	0.776	16.794	0.06	<0.001
	CP 8	0.964	0.751	16.145	0.06	<0.001
Psychological ownership	PO 1	1	0.777	-	-	-
	PO 2	0.933	0.765	15.437	0.06	<0.001
	PO 3	0.972	0.784	15.848	0.061	<0.001
	PO 4	0.927	0.78	15.758	0.059	<0.001
Purchase intention	PI 1	1	0.804	-	-	-
	PI 2	1.008	0.813	16.592	0.061	<0.001
	PI 3	0.978	0.797	16.298	0.06	<0.001

**Table 10:** Model path coefficients.

Hypothesis	Path	Unstandardized Coefficients	Standardized Coefficients	Standard Error	*z*	*p*	Results
H1a	UQ → PI	0.221	0.189	0.077	2.882	0.004	Supported
H1b	UE → PI	0.203	0.184	0.07	2.881	0.004	Supported
H1c	UI → PI	0.19	0.17	0.07	2.721	0.007	Supported
H2	PO → PI	0.179	0.176	0.071	2.517	0.012	Supported
H3a	UQ → PO	0.278	0.243	0.072	3.867	<0.001	Supported
H3b	UE → PO	0.127	0.117	0.067	1.891	0.059	Supported
H3c	UI → PO	0.23	0.211	0.066	3.486	<0.001	Supported
H4	CP → PI	0.179	0.164	0.067	2.665	0.008	Supported
H5a	UQ → CP	0.237	0.221	0.067	3.517	<0.001	Supported
H5b	UE → CP	0.218	0.215	0.064	3.391	0.001	Supported
H5c	UI → CP	0.301	0.294	0.062	4.824	<0.001	Supported

**Table 11:** Mediating effect test (communication proximity).

Path	Mediating Effect Value	Bootstrap (5000 Times)			95%BootCI
		Boot SE	*z*-Value	*p*-Value	
UGC quality → Communication proximity → Purchase intention	0.037	0.014	2.66	0.008	0.015~0.071
UGC emotion → Communication proximity → Purchase intention	0.043	0.016	2.627	0.009	0.017~0.081
UGC interaction → Communication proximity → Purchase intention	0.058	0.019	3.115	0.002	0.027~0.1

**Table 12:** Mediating effect test (psychological ownership).

Path	Mediating Effect Value	Bootstrap (5000 Times)			95%BootCI
		Boot SE	*z*-Value	*p*-Value	
UGC quality → Psychological ownership → Purchase intention	0.062	0.017	3.588	<0.001	0.024~0.091
UGC emotion → Psychological ownership → Purchase intention	0.041	0.014	2.901	0.004	0.012~0.068
UGC interaction → Psychological ownership → Purchase intention	0.064	0.018	3.535	<0.001	0.026~0.097

## Data Availability

The raw data supporting the conclusions of this article will be made available by the authors on request.
